# Characteristics of headache attributed to COVID-19 infection and predictors of its frequency and intensity: A cross sectional study

**DOI:** 10.1177/0333102420965140

**Published:** 2020-11-04

**Authors:** Rehab Magdy, Mona Hussein, Christine Ragaie, Hoda M Abdel-Hamid, Ahmed Khallaf, Hoda I Rizk, Ahmed Dahshan

**Affiliations:** 1 Department of Neurology, Cairo University, Cairo, Egypt; 2 Department of Neurology, Beni-Suef University, Beni-Suef, Egypt; 3 Public Health and Community Medicine, Cairo University, Cairo, Egypt

**Keywords:** COVID-19 related headache, primary headache disorders, VAS, fever, dehydration

## Abstract

**Objective:**

To study the characteristics of headache attributed to COVID-19 infection and predictors of its severity.

**Methods:**

A cross-sectional study involved 172 individuals who had headache due to COVID-19 infection. A detailed analysis of such headache was done through a face-to-face interview. Patients with any other form of secondary headache were excluded. Labs, including lymphocytic count, C-reactive protein, D-dimer and ferritin and chest imaging, were made available.

**Results: The:**

majority of our patients had a diffuse headache (52.9%). It was pressing in 40.7%, with median intensity of 7 (assessed by visual analogue scale) and median frequency of 7 days/week. Patients with preexisting primary headache (52.9%) had significantly more frequent COVID-19 related headache than those without (47.1%) (*p* = 0.001). Dehydrated patients (64.5%) had more frequent COVID-19 related headache than those who were not dehydrated (35.5%) (*p* = 0.029). Patients with fever (69.8%) had significantly higher frequency and intensity of COVID-19 related headache compared to those without fever (30.2%) (*p* = 0.003, 0.012). Patients with comorbidities (19.8%) had significantly higher frequency and intensity of headache than those without comorbidities (80.2%) (*p* = 0.006, 0.003). After multiple linear regression, primary headache disorders, dehydration and comorbidities were considered predictors of frequency of COVID-19 related headache. Meanwhile, fever and dehydration were predictors of pain intensity.

**Conclusion:**

Healthcare providers of COVID-19 patients need to be aware of frequency and intensity predictors of COVID-19 related headache: Primary headache disorders, fever, dehydration, and comorbidities.

## Introduction

Coronavirus disease 2019 (COVID-19) is an escalating crisis all over the world caused by severe acute respiratory syndrome coronavirus 2 (SARS-CoV-2) (1). It was first identified in December 2019 in Wuhan, China (2). The World Health Organization (WHO) declared the 2019–20 coronavirus outbreak a Public Health Emergency of International Concern (PHEIC) on 30 January 2020, and a pandemic on 11 March 2020 (3,4).

Common symptoms include headache, fever, malaise, muscle and joint pains, dyspnea, cough, and loss of smell and taste (5,6). Less common symptoms include abdominal pain, nausea, vomiting and diarrhea (7). The time from exposure to onset of symptoms ranges from 5–14 days (8). While the majority of cases have mild symptoms, some cases may progress to acute respiratory distress syndrome (ARDS).The highest proportion of severe cases occurs in adults over 60, and in those with certain underlying chronic co-morbidities such as diabetes, cerebrovascular and cardio-vascular diseases (9,10).

Multiple studies confirmed that headache was a frequently reported symptom in patients infected with SARS-COV-2. However, there was a great diversity in its frequency, severity, character and duration (11). Borges do Nascimento et al. (12) found that headache was the most common neurological symptom in patients with COVID, as it was observed in 12% of confirmed cases. In another study, headache was reported to be the second most common neurological symptom after dizziness and was present in 13% of the patients (13).

Much concern was directed towards clarifying the criteria of headache attributed to systemic infection. Headache is usually diffuse and bilateral, but in some cases, it may be fronto-temporal or occipital with associated retroocular pain. The intensity of headache is variable and it may increase by coughing, straining or head movement. It may be associated with conjunctival injection, nausea, vomiting, photophobia or phonophobia. Fever was considered one of the most significant predictors of frequency and intensity of such a type of headache (14).

In some susceptible individuals, systemic infections may be a provoking factor for an underlying primary headache disorder. Consequently, caution must be given when trying to distinguish whether a patient’s headache is an infection triggering pre-existing primary headache attack, or is an acute headache attributable to a systemic viral infection (14).

The International Classification of Headache Disorders, third edition (ICHD-3) requires a confirmed diagnosis of systemic viral infection in the absence of meningitic or encephalitic involvement for the diagnosis of headache attributed to systemic viral infections (15).

The aim of this work was to study the headache characteristics in patients infected with COVID-19, and to detect predictors of its frequency and intensity in relation to history of pre-existing primary headache disorder, medical comorbidities, fever, dehydration, steroids intake, laboratory biomarkers and severity of chest imaging findings of SARS-COV-2 infection.

## Methods

### Study design and participants

This is a cross-sectional study, carried out on 172 patients suffering from COVID-19 related headache in the period from 1 April 2020 to 1 June 2020. Patients were recruited from two quarantine hospitals in two Egyptian governorates, Cairo and Beni-Suef.

The diagnosis of COVID-19 infection and grading of its severity were made according to the clinical management of COVID-19, released by the World Health Organization (WHO) (16). The polymerase chain reaction (PCR) testing of a nasopharyngeal sample should have tested positive for COVID-19.

The grading of COVID-19 severity was stratified into mild, moderate, or severe according to WHO classification (16) as follows: Mild COVID-19: Patients with COVID-19 infection without evidence of viral pneumonia or hypoxia. Moderate COVID-19: Patients with clinical signs of pneumonia (fever, cough, dyspnea, tachypnea) but no signs of severe pneumonia, including SpO_2_ ≥90% on room air. Severe COVID-19: Patients with clinical signs of pneumonia plus one of the following: Respiratory rate >30 breaths/min; severe respiratory distress; or SpO_2_ < 90% on room air (16).

The treatment protocol used in the two centers was in accordance with the treatment guidelines for national institutes of health (NIH); the nation's medical research agency (17) steroids were used in treatment protocol of moderate cases that required supplemental oxygen and in severe cases that required mechanical ventilation.

Any adult patient (age ≥18 years) suffering from headache related COVID-19 infection according to the ICHD-III criteria of acute headache attributed to systemic viral infection (9.2.2.1) (15) was eligible. Patients with any other form of secondary headache (from detailed neurological and medical history, fundus examination or brain imaging) were excluded. Patients with evidence of intracranial infections (from history, neurological examination, brain imaging or CSF analysis), or patients with altered mental state, structural lesion in brain imaging, or participating in clinical trials during the recruitment period were excluded.

**Figure 1.**
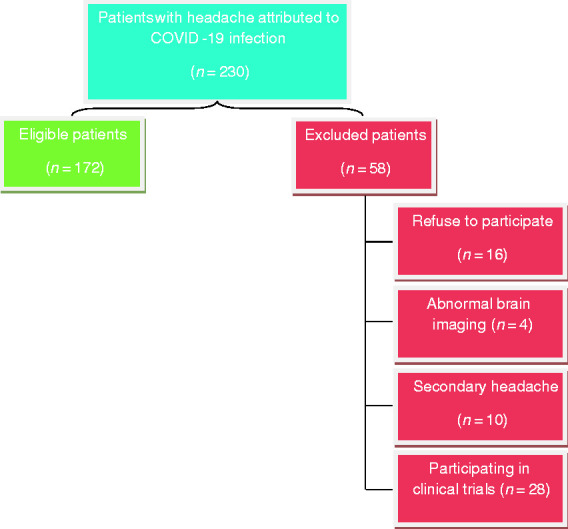
Flow diagram for the eligible and excluded patients.

Out of 230 patients with headache due to COVID-19, 58 were excluded (16 declined to participate, four had abnormal brain imaging, 10 had secondary headache and 28 were participating in other clinical trials) (Figure 1).

### Data collection

We collected data on patient demographics, body mass index (BMI) and comorbid conditions. In addition to baseline clinical data, an expert neurologist made a detailed descriptive analysis of COVID-19 related headache by face-to-face interviews. A stepwise approach was applied to see if there was pre-existing primary headache disorder according to the ICHD-3 criteria (15). COVID-19 related headache analysis data included site of headache, frequency, duration, character, intensity according to the visual analogue scale (VAS) (18), and response to analgesics. The onset of headache in temporal relation to other COVID-19 symptoms was recorded. The relation of headache to administration of steroids, periods of high fever and dehydration, if present, were also reported.

According to the 2015 Cochrane review (19), dehydration was defined as serum osmolality of more than 294 mOsm/kg in the context of water loss. Serum osmolality was calculated using the following formula: 2 Na (mEq/L) + (Urea [mg/dL])/2.8 + (Glucose [mg/dL])/18.

Laboratory data, including baseline serum C-reactive protein (CRP), D-dimer, ferritin and lymphocytic count done within 24 hours of hospital admission, were collected. The results of initial computerized tomography (CT) – chest were recorded.

### Sampling

All patients with COVID-19 related headache who were admitted to the two centers during the recruitment period that fulfilled the eligibility criteria and were accepted to take part in the study were included in the study (172 patients).

### Statistical analysis

The data were analyzed using IBM SPSS (Statistical Package of Social Science) Version 21. Normality distribution of the data was tested by using the Shapiro-Wilk test. Clinical characteristics and laboratory data of the patients, in addition to the characteristics of COVID-19 related headache, were presented by using the median and interquartile range (IQR) for non-normally distributed numerical data and by using frequency and percentage for qualitative data. The associations of intensity, frequency, and duration of headache with characteristics of the patient were assessed through χ^2^ tests for categorical variables and through Mann-Whitney and Kruskal-Wallis tests for continuous variables (with results non-normally distributed according to the Shapiro-Wilk test). Multivariate analysis was done with construction of a multiple linear regression model to identify predictors of frequency and intensity of COVID-19 related headache after being adjusted for their potential mutual confounding effect. We did not include in this model factors that showed collinearity; we analysed multicollinearity by using the variance inflation factor (VIF) and tolerance. We considered multicollinearity critical when VIF was >3 and tolerance less than 0.1. The magnitude of the dependence between the variables was explored using the adjusted R^2^ value. The results are presented as odds ratios (OR) and 95% confidence intervals (CIs). A *p*-value less than 0.05 was considered statistically significant. All tests were two-tailed.

### Ethical considerations

The study proposal was revised and approved by the Neurology Department Ethical Committee, Cairo University. The aim and nature of the study were explained for each patient before inclusion. A policy of data confidentiality was firmly followed. Informed written consent was obtained from all participants before enrolment. The study design followed the requirements of the Revised Helsinki Declaration of biomedical ethics.

## Results

### Demographics and clinical characteristics of the study population

The study population included 172 patients who had headache attributed to COVID-19 infection, with median age of 33 and interquartile range (IQR) 27.3–42 years. The study included 64 males (37.2%) and 108 females (62.8%).Other clinical characteristics and laboratory findings of the study group are summarized in Table 1. The characteristics of COVID-19 related headache are outlined in Table 2.

**Table 1.** Demographics, clinical characteristics and laboratory data of the study group.

Patients (n = 172)	
Age, median (IQR)	33 (27.3–42)
Gender, n (%)
Males	64 (37.2%)
Females	108 (68.2%)
BMI, median (IQR)	27.7 (25.4–31.6)
Smokers, n (%)	15 (8.7%)
Medical comorbidities*, n (%)	34 (19.8%)
Preexisting primary headache disorder, n (%)
Migraine	45 (26.2)
Tension-type headache	46 (26.7)
None	81 (47.1%)
Laboratory data, median (IQR)
Lymphocyte count	1400 (800–2447.5)
CRP (mg/L)	12 (4.39–48)
D-dimer (µg/mL)	0.36 (0.2–0.56)
Ferritin (ng/mL)	130 (52.1–261.5)
Grading severity of COVID-19 infection, n (%)
Mild	116 (67.4%)
Moderate	42 (24.4%)
Severe	14 (8.1%)

*Eighteen patients with controlled HTN (BP ≥ 140/90 or on antihypertensive medications), seven patients were diabetic, five patients had hypothyroidism, three patients had ischemic heart disease and one patient had epilepsy.

BMI: body mass index; CRP: C-reactive protein; IQR: interquartile range.

**Table 2.** Characteristics of COVID-19 related headache.

Headache characteristics	Patients (n = 172)
Headache onset, n (%)
Before other COVID-19 symptoms	49 (28.5%)
With other COVID-19 symptoms	98 (57%)
After other COVID-19 symptoms	25 (14.5%)
Site, n (%)
Diffuse	91 (52.9%)
Temporal	31 (18%)
Frontal	40 (23.3%)
Occipital	10 (5.8%)
Character of pain, n (%)
Throbbing	28 (16.3%)
Pressing	70 (40.7%)
Exploding	45 (26.2%)
Dull	29 (16.9%)
Frequency (attacks/week), median (IQR)	7 (3–7)
Visual analogue scale, median (IQR)	7 (5–8)
Duration in hours, median (IQR)	6 (2–16 )
Relation to high fever*, n (%)
Increase with fever	59 (49.2%)
Decrease with fever	4 (3.3%)
No effect	57 (47.5%)
Relation to steroids intake**, n (%)
Increases with steroids	9 (16.7%)
Decreased with steroids	22 (40.7%)
No effect of steroids	23 (42.6%)
Relation to dehydration***, n (%)
Increased with dehydration	30 (27%)
Decreased with dehydration	12 (10.8%)
No effect of dehydration	69 (62%)
Response to analgesics, n (%)
Excellent	56 (32.6%)
Moderate	80 (46.5%)
Poor	36 (20.9%)

*Only 120 (69.7%) patients had fever.

**Only 54 (31.4%) of patients received steroids.

***Only 111 (64.5%) patients had dehydration.

IQR: interquartile range.

### Characteristics of COVID-19 related headache in relation to pre-existing primary headache disorder

**Figure 2.**
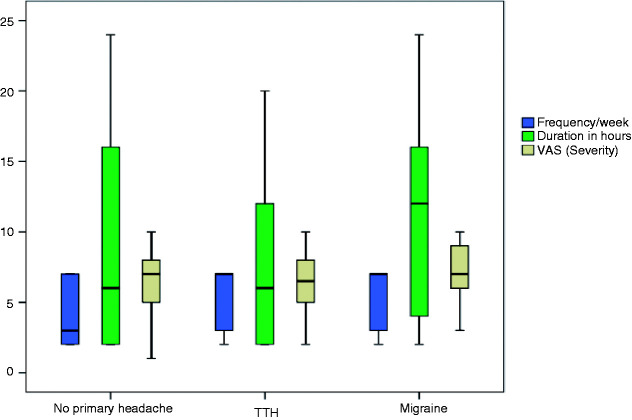
Comparison between patients with migraine, tension type headache and patients without primary headache, regarding characteristics of COVID-19 related headache.

Patients having a pre-existing primary headache disorder had significantly more frequent COVID-19 related headache (*p* = 0.003) (Table 3). Frequency of COVID-19 related headache was significantly different between the three groups: Patients with migraine, patients with tension-type headache (TTH) and patients without pre-existing primary headaches (Figure 2). *Post hoc* pairwise comparisons of the Kruskal-Wallis test showed that such frequency was significantly higher in patients with migraine and TTH compared to patients without pre-existing primary headache, (*p* < 0.001 and *p* = 0.011), respectively.

**Table 3.** Characteristics of COVID-19 related headache in relation to gender, smoking state, pre-existing primary headache disorder, fever, dehydration, steroids, comorbidities and COVID-19 severity.

	Frequency/week, median (IQR)	Duration (hours), median (IQR)	VAS, median (IQR)
Gender
Males, n = 64 (37.2%)	3 (2–7)	16 (2–24)	7 (0–10)
Females, n = 108 (62.8%)	5 (2–7)	12 (2–24)	8 (0–10)
*p*-value	0.56	0.54	0.51
Smoking state
Smoker, n = 15 (8.7%)	6 (2–7)	16 (2–24)	7 (4–10)
Non-smoker, n = 157 (91.2 %)	4 (2–7)	10 (2–24)	5 (0–10)
*p-*value	0.45	0.36	0.77
Pre-existing primaryheadache*
With, n = 91 (52.9%)	7 (3–7)	12 (4–16)	7 (5–8)
Without, n = 81 (47.1%)	3 (2–7)	6 (2–16)	7 (5–8)
*p*-value	<0.001*	0.55	0.34
Fever*
With, n = 120 (69.8%)	7 (3–7)	11 (4–16)	7 (6–8)
Without, n = 52 (30.2%)	3 (2–7)	6 (2–16)	6 (4–8)
*p*-value	0.003*	0.292	0.012*
Dehydration*
With, n = 111 (64.5%)	7 (3–7)	6 (2–16)	7 (5–8)
Without, n = 61 (35.5%)	3 (2–7)	6 (2–16)	6 (5–8)
*p*-value	0.029*	0.506	0.201
Steroids
Received, n = 54 (31.4%)	7 (3–7)	6 (2–13)	7 (5–8)
Not received, n = 118 (68.6%)	7 (3–7)	6 (4–16)	7 (5–8)
*p*-value	0.965	0.295	0.924
Comorbidities
With, n = 34 (19.8%)	7 (7–7)	12 (2–16)	8 (6–10)
Without, n = 138 (80.2%)	5 (2–7)	6 (2–16)	6.5 (5–8)
*p*-value	0.006*	0.235	0.003*
Grading severity of COVID-19 infection
Mild, n = 116 (67.4%)	3 (2–7)	6 (2–15)	6 (5–8)
Moderate, n = 42 (24.4%)	7 (3–7)	12 (2–16)	8 (6.75–9)
Severe, n = 14 (8.1%)	7 (2.75–7)	6 (2–16)	7 (5–8.25)
*p*-value	0.032*	0.134	0.007*

VAS: visual analogue scale.

Note: Bold *p*-values < 0.05 are considered significant.

*These groups were matched regarding age, sex, comorbidities and COVID-19 severity.

### Characteristics of COVID-19 related headache in relation to other COVID-19 symptoms

Patients with fever had significantly higher frequency and intensity of COVID-19 related headache compared to patients without fever. Headache frequency was significantly higher in patients with dehydration in comparison to those without dehydration (Table 3).

### Characteristics of COVID-19 related headache in relation to comorbidities, gender and smoking status of the patients

Patients with comorbidities had significantly higher frequency and intensity of COVID-19 related headache compared to patients without comorbidities (Table 3). Nevertheless, headache characteristics did not differ in terms of gender or smoking status.

### Characteristics of COVID-19 related headache in relation to severity of COVID-19 infection

Each of frequency and intensity of COVID-19 related headache was significantly different between the three groups (Table 3). *Post hoc* pairwise comparisons of the Kruskal-Wallis test showed that frequency and intensity were significantly higher in patients with moderate COVID-19 than in those with mild COVID-19 infection (*p* = 0.031, *p* = 0.005), respectively.

### Factors associated with frequency and intensity of COVID-19 related headache

A multivariate analysis was done including all variables that were believed to be relevant for frequency and intensity of COVID-19 headache, and these showed no collinearity with each other (VIF < 3).

Factors associated with high frequency of attacks of COVID-19 related headache were the presence of primary headache group disorders (*p* = 0.026), dehydration (*p* = 0.007) and comorbidities (*p* = 0.026), (adjusted R^2^, 0.28) (Table 4).

**Table 4.** Factors related to frequency of COVID-19 related headache, multivariate analysis.

Variables	B	Odds ratio	Sig.	95.0% confidence interval for B		Correlations			Collinearity statistics	
				Lower bound	Upper bound	Zero-order	Partial	Part	Tolerance	VIF
Constant	3.528		0.042	0.125	6.931					
Lymphocyte count	−0.005	−0.016	0.892	−0.001	0.000	−0.037	−0.019	−0.014	0.807	1.239
CRP (mg/L)	0.003	0.062	0.650	−0.010	0.016	0.314	0.063	0.048	0.607	1.647
D-dimer	0.220	0.041	0.741	−1.109	1.548	0.142	0.046	0.035	0.742	1.348
Ferritin (ng/mL)	−0.005	−0.015	0.914	0.001	0.001	0.178	−0.015	−0.011	0.563	1.776
Primary headache	1.437	0.314	0.026*	0.182	2.692	0.296	0.304	0.243	0.599	1.669
Dehydration	1.586	0.334	0.007*	0.444	2.728	0.463	0.361	0.294	0.775	1.290
Fever	0.794	0.149	0.228	−0.513	2.102	0.202	0.167	0.129	0.745	1.342
Moderate COVID-19	0.231	0.045	0.721	−1.057	1.519	0.150	0.050	0.038	0.711	1.406
Severe COVID-19	−0.666	−0.095	0.528	−2.768	1.436	0.051	−0.088	−0.067	0.497	2.011
Age	−0.031	−0.175	0.248	−0.084	0.022	0.044	−0.160	−0.123	0.495	2.021
Sex	−0.284	−0.061	0.670	−1.612	1.045	0.018	−0.059	−0.045	0.549	1.823
Smoking	0.686	0.072	0.554	−1.626	2.999	0.090	0.082	0.063	0.769	1.301
Comorbidities	2.055	0.394	0.026*	0.256	3.853	0.304	0.303	0.242	0.378	2.644
Dependent variable: Frequency/week, adjusted R^2^ 0.28.										

CRP: C-reactive protein; VIF: variance inflation factor.

Note: Bold *p*-values < 0.05 are considered significant.

Factors associated with high pain intensity of COVID-19 related headache were female gender (*p* = 0.006), dehydration (*p* = 0.024) and fever (*p* = 0.046), (adjusted R^2^, 0.3) (Table 5).

**Table 5.** Factors related to intensity of COVID-19 related headache, multivariate analysis.

Variables	B	Odds ratio	Sig	95.0% confidence interval for B		Correlations			Collinearity statistics	
				Lower bound	Upper bound	Zero-order	Partial	Part	Tolerance	VIF
Constant	0.429		0.803	−3.002	3.860					
Lymphocyte count	0.000	0.065	0.577	0.000	0.001	−0.025	0.078	0.058	0.807	1.239
CRP (mg/L)	0.000	−0.004	0.976	−0.013	0.013	0.281	0.004	−0.003	0.607	1.647
D-dimer	0.306	0.055	0.649	−1.034	1.645	0.172	0.063	.048	0.742	1.348
Ferritin (ng/mL)	0.001	0.206	0.144	0.000	0.002	0.221	0.202	0.154	0.563	1.776
Primary headache	0.097	0.021	0.878	−1.168	1.363	0.124	0.021	0.016	0.599	1.669
Dehydration	1.332	0.274	0.024*	0.180	2.483	0.292	0.306	0.241	0.775	1.290
Fever	1.346	0.247	0.046*	0.027	2.664	0.339	0.273	0.213	0.745	1.342
Moderate COVID-19	0.587	0.112	0.369	−0.711	1.885	0.237	0.125	0.094	0.711	1.406
Severe COVID-19	0.031	0.004	0.977	−2.088	2.150	0.115	0.004	0.003	0.497	2.011
Age	−0.010	−0.057	0.700	−0.064	0.043	0.141	−0.054	−0.040	0.495	2.021
Sex	1.897	0.399	0.006*	0.558	3.236	0.252	0.367	0.296	0.549	1.823
Smoking	1.724	0.176	0.144	−0.608	4.055	0.119	0.202	0.154	0.769	1.301
Comorbidities	1.119	0.209	0.221	−0.694	2.932	0.377	0.169	0.129	0.378	2.644
Dependent variable: VAS (intensity), adjusted R^2^ 0.3.										

CRP: C-reactive protein; VAS: visual analog scale; VIF: variance inflation factor.

Note: Bold *p*-values < 0.05 are considered significant.

## Discussion

Clinicians involved in the care of COVID-19 patients need to be cognizant of the diverse clinical characteristics of COVID-19 related headache. This is the first study that offers a detailed analysis of COVID-19 related headache in relation to history of pre-existing primary headache disorder, medical comorbidities, fever, dehydration, intake of steroids and laboratory biomarkers of SARS-COV-2 infection.

In general, the headaches that accompany systemic infections are typically nonspecific without any particular distinguishing or characteristic features (20). Although diffuse pain and moderate or severe intensity are listed in the ICHD-3 criteria of “Acute headache attributed to systemic viral infection”, they are not mandatory for diagnosis (15). The present study showed that the majority of our patients had a diffuse headache (52.9%), pressing in 40.7%, with median intensity of 7.

Pathogenesis of headaches attributed to systemic infection is not clearly settled. It has been speculated that microorganisms may activate inflammatory and nociceptive mediators that stimulate headache, such as nitric oxide, prostaglandins and cytokines (14,21). Likewise, COVID-19 infection is accompanied by release of a large amount of pro-inflammatory cytokines like IL-1β, IL-6, and TNF-α (22) that are involved in various pathological pain states (23). The response to steroids in 40.7% of our patients who received steroids may be indicative of immunological/inflammatory mechanisms for the generation of COVID-19 related headache. However, we did not find any statistically significant relationship between the use of steroids and frequency, duration or intensity of COVID-19 related headache.

There is another point of view: Fever, as a part of a systemic infection, may be a trigger for headache (14,19). The principal inflammatory mediators of SARS-COV-2 infection (IL1 and IL6) activate the hypothalamus and promote fever (24). In accordance with our results, COVID-19 patients with fever were more likely to develop more frequent COVID-19 related headache attacks, compared to patients without fever. Nevertheless, we should not rely on this principle alone, as some patients (30.2%) had headache in the absence of fever.

The present study showed that patients with pre-existing primary headache disorder had significantly more frequent attacks of COVID-19 related headache. Pre-existing primary headache disorder is a demodulatory pain process (25), where derangement of top-down pain modulatory pathways occurs with atypical release of nociceptive molecules (26). Such alterations can lead to sensitization of central and peripheral nociceptive pathways resulting in a decrease in the pain threshold and an increase in receptive fields (27,28). Specifically for migraine, cortical spreading depression (CSD) may add to hyperexcitability of the trigeminovascular neurons (29).

The current study showed that dehydration is a predictor of higher frequency and intensity of COVID-19 related headache attacks. It is well known that water deprivation triggers migraine and other types of headache due to intracranial dehydration and total plasma volume (30–32. Therefore, healthcare providers must be aware of fluid balance states in patients with COVID-19, especially for those with headache.

Medical comorbidities are considered some of the most evident predictors of COVID-19 severity (33). Although patients with and without comorbidities in our study were not matched regarding COVID-19 severity, we could not neglect the significant impact of comorbidities and both frequency and intensity of headache revealed by the regression model. Furthermore, medical comorbidities were indicated by multiple researchers as risk factors for headache (34–36).

To our knowledge, laboratory markers of COVID-19 infection were studied for the first time in relation to frequency and intensity of COVID-19 related headache. It is well known that higher CRP is related to decreased pain threshold (37) and is implicated in the inflammatory process of various types of headache (38). However, in our study, CRP was not found to be associated with either frequency or intensity of COVID-19 related headache

Lymphocytes are the primary sources of proinflammatory and anti-inflammatory cells that are strongly involved during the systemic inflammatory response (39). Karabulut et al. (40) found a significantly higher neutrophil/lymphocyte ratio during the migraine attacks, compared to the healthy subjects. However, in our study, lymphocytic count was not found to be associated with either frequency or intensity of COVID-19 related headache.

Although the process of iron metabolism is supposed to increase the frequency of headaches by decreasing the pain threshold via different mechanisms – such as nitric oxide and neurotransmitters (40) – our study failed to find such a relation. Likewise, Aamodt et al. (41) found no association between ferritin levels and the prevalence of headaches.

D-dimers, as fibrin degradation products, are commonly used markers of inflammation and increased coagulation activity (42). Although higher D-dimer levels were supposed to play a role in neurogenic inflammation in migraine (43), we did not find any relation with frequency or intensity of COVID-19 related headache.

The most important limitation of our study is the low number of patients with severe COVID-19 infection (n = 14) and patients who received steroids (n = 54). As a result, we could not properly judge the impact of COVID-19 severity and intake of steroids on the frequency, intensity and duration of COVID-19 related headache. Secondly, we did not compare between the impact of different medical comorbidities on frequency, intensity and duration of COVID-19 related headache because the included subgroups (HTN, DM, hypothyroidism, ischemic heart disease and epilepsy) did not have comparable numbers of patients. Future studies should adopt follow-up of patients who have recovered from SARS-COV-2 infection to assess the possibility of development of chronic post-infection headache. In addition, much concern should be directed towards clarifying the pathogenesis of COVID-19 related headache.

## Conclusion

COVID-19 related headache is diffuse in the majority of patients (52.9%), pressing in 40.7%, with a median intensity of 7 (as assessed by VAS) and a median frequency of 7 days/week. Frequency of COVID-19 related headache was higher in patients with primary headache group disorders, dehydration and comorbidities. Meanwhile, high pain intensity was associated with female gender, fever, and dehydration.

## Clinical implications

- COVID-19 related headache is diffuse, pressing in the majority of patients, with moderate to severe intensity.
- Patients with a history of pre-existing primary headache disorders, comorbidities, or dehydration had more frequent COVID-19 related headache attacks.
- Fever, dehydration and female gender are predictors of higher intensity of COVID-19 related headache.
